# Plant Host-Associated Mechanisms for Microbial Selection

**DOI:** 10.3389/fpls.2019.00862

**Published:** 2019-07-03

**Authors:** Piet Jones, Benjamin J. Garcia, Anna Furches, Gerald A. Tuskan, Daniel Jacobson

**Affiliations:** ^1^Oak Ridge National Laboratory, Biosciences Division, The Center for Bioenergy Innovation, Oak Ridge, TN, United States; ^2^The Bredesen Center for Interdisciplinary Research and Graduate Education, University of Tennessee, Knoxville, Knoxville, TN, United States

**Keywords:** microbial community, jasmonic acid, salicylic acid, ethylene, keystone species, abiotic stress, biotic stress, microbiota

## Abstract

Plants serve as host to numerous microorganisms. The members of these microbial communities interact among each other and with the plant, and there is increasing evidence to suggest that the microbial community may promote plant growth, improve drought tolerance, facilitate pathogen defense and even assist in environmental remediation. Therefore, it is important to better understand the mechanisms that influence the composition and structure of microbial communities, and what role the host may play in the recruitment and control of its microbiome. In particular, there is a growing body of research to suggest that plant defense systems not only provide a layer of protection against pathogens but may also actively manage the composition of the overall microbiome. In this review, we provide an overview of the current research into mechanisms employed by the plant host to select for and control its microbiome. We specifically review recent research that expands upon the role of keystone microbial species, phytohormones, and abiotic stress, and in how they relate to plant driven dynamic microbial structuring.

## 1. Introduction

**Figure 1:**
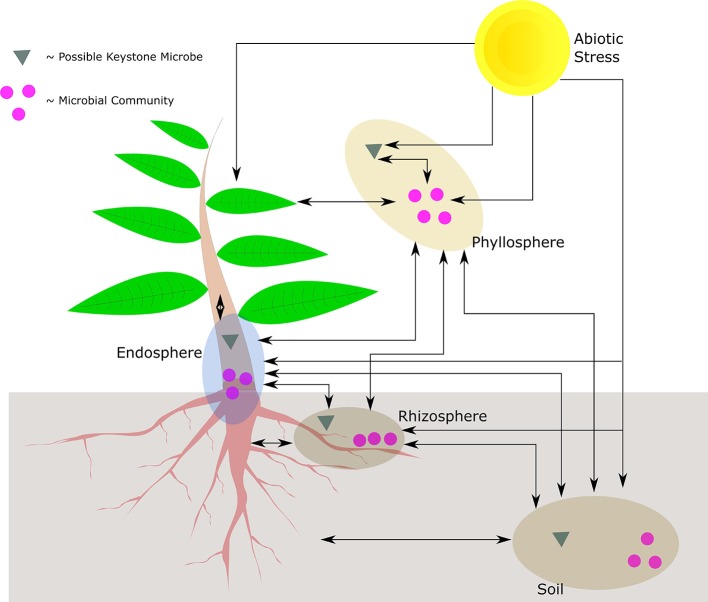
Possible interactions in the phytobiome between the plant, abiotic stress, keystone microbes, and microbial communities. Illustrated are the respective compartments of the holobiont, only the phyllosphere indicates keystone microbial interaction though the other compartments may have the same type of interactions. The phyllosphere may also be epithytic or endophytic. The cross-interaction between different compartments may be mediated at a community level, or by means of individual (keystone) microbes. In addition, the plant may also interact with whole communities or by means of individual microbes.

The sessile nature of plants limits their capacity to deal with an immediate and localized disturbance, irrespective of whether the disturbance is caused by biotic or abiotic stress. It therefore stands to reason that plants have evolved systems to manage the impact of these collective and respective stresses. From a biotic microbial view point, plants play host to a number of organisms that reside in the phyllosphere, endosphere, and rhizosphere, influencing how a plant reacts to its environment. If viewed in the context of an ecological unit, the community of organisms is known as the holobiont. Further incorporating the environment results in what is collectively known as the phytobiome, where the possible plant-microbe-stress interactions are given in Figure 1.

The holobiont has a much greater evolutionary potential for dealing with biotic and abiotic stress than the plant itself. Therefore, it is potentially more sustainable to manage abiotic/biotic stresses in a holistic and multifaceted manner. The plant employs a combinatorially complex system of receptors and signals to adapt to different stressors (Hacquard et al., 2017). Unraveling the complexity of the system is not a trivial task, with researchers providing different perspectives for elucidating a contextual understanding of the dynamics of plant-microbiome interaction.

The improved understanding of the interactions between the plant and its microbiome has broadened our knowledge on the capabilities of the plant to influence its microbiome and vice versa. In interacting with its microbiome, plants have the capacity to release chemical signals into their environment. The signals can either have a positive or negative effect on other plants or members of the microbiome. Root exudates, comprised of allelochemicals, have been associated with signaling in plant-microbe interaction and can also facilitate plant to plant communication (Bais et al., 2004). Exudates with potential allelopathic properties can help the plant both positively and negatively select for members of their phytobiome (Bertin et al., 2003; Sasse et al., 2018), allowing the plant to establish a rhizosphere and soil microbiome that may also be beneficial or detrimental to other plants and microbes. The concept of influencing the plant phytobiome has also been explored in biocontrol strategies, e.g., strategies against nematodes (Stirling, 2017). The ability of the plant, together with individual members of its microbiome, to control and shape the overall microbiome influences a plant's growth and stress response. A better understanding of the resultant interplay between defense and control may allow for an optimized holobiont that can benefit, among others, agricultural and bioremediation efforts (Ojuederie and Babalola, 2017; Pappas et al., 2017; Ab Rahman et al., 2018).

**Figure 2:**
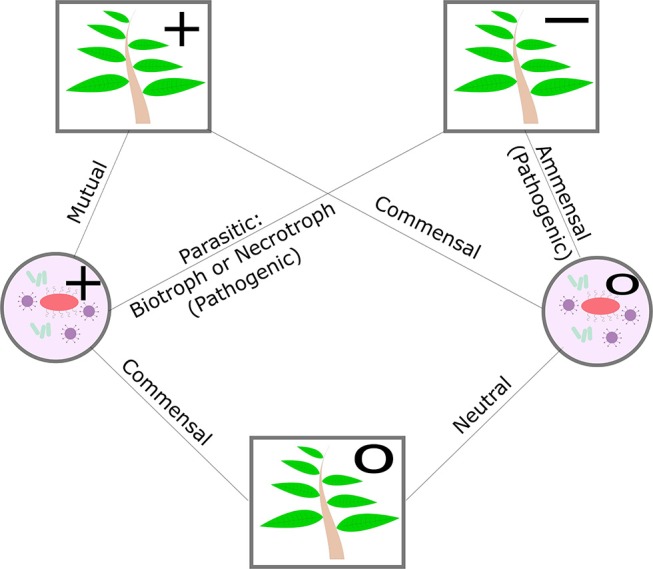
Diagram of plant-microbe symbiosis, excluding negative effects on microbes. Circle nodes in the diagram indicate microbes, square nodes indicate the plant. Positive effects are indicated with “+,” negative effects with a “−,” and neutral effects by a “o”.

The microbes that a plant hosts are broadly classified as pathogenic or non-pathogenic. The nature of the non-pathogenic interaction may be beneficial, mutualistic, commensal, or neutral and pathogenic interactions may be parasitic or amensal. The plant can play host to biotrophs, who receive nourishment from a living host cell, necrotrophs, who receive nourishment from a dead host cell, and hemitrophs, who switch between the different nourishment states (see Figure 2). The nature of plant-microbe relationships and the ability of the plant to influence select neighbors may potentially benefit the plant's own growth or defense (Bulgarelli et al., 2013; Mikiciński et al., 2016). Nutritional deficiencies in the plant can also alter the transcriptional profile of its microbiome and thereby mitigate the impact of nutritional stress (Carvalhais et al., 2013). Additionally, the plant's abiotic stress tolerance and disease resistance may not be a mutually exclusive processes. For example, common mechanisms exist between phytohormone-based pathogen defense responses and the plant's ability to tolerate drought and salt stress (Cho et al., 2013; Huang et al., 2018). At a community level, evidence suggests that the ability of the plant host to shape its microbial community may also serve as an additional layer of defense to disease and stress (Hacquard et al., 2017; Berendsen et al., 2018).

Interaction between plants and microbes has generally been studied with respect to individual (or small collections of) microbes. Additionally, the focus has often been on how the microbe negatively or positively impacts the plant and not how a plant may influence the selection of microbes under biotic or abiotic stress. However, there is dynamic feedback between a plant and a microbe that may impact the plant's microbial community (Bever et al., 2012). The reciprocal nature of the interactions between the plant and its microbial community is poorly understood. It is therefore not only important to determine how individual microbes may impact the plant, but also how the plant may impact its community. There is also evidence to suggest that the plant itself may not be the only determinant in microbial diversity, suggesting the environment and abiotic factors are also important to consider when identifying plant-microbial interactions (Kennedy et al., 2004; Whitaker et al., 2018). There is still much that we can learn about the interplay between the different elements of the phytobiome, including (1) how do the individual microbes influence the plant-mediated structure, (2) how can the plant shape its microbiome through signaling and nutrient availability, and (3) what role does the abiotic stress play in the plant's ability to influence microbial community members and structure? We review recent contributions to the understanding of the impact that keystone members of the microbiome may have on the plant and other community members. We also review the current understanding of the extent to which a plant may shape its microbial community. Finally we look at how abiotic stress on the plant influences the microbial community. Each of the mechanisms that a plant employs to interact with its microbial community and abiotic stresses is part of a complex dynamic system that influences plant growth and stress tolerance.

## 2. Keystone Microbial Species

Improving our ability to manipulate plant phenotypes, including growth, is of great interest for agricultural, industrial, and ecological restoration efforts, among others. Understanding the mechanisms that underpin pathogen resistance, abiotic stress tolerance, and range shifts (including the ability to establish crops in marginal agricultural land) is of particular importance in the face of shifting climate conditions. An important mechanism through which a plant shapes its microbiome and larger ecosystem is through interactions with keystone microbial species. The concept of keystone species was developed by ecologist Robert T. Paine (Paine, 1966, 1969) and has since been applied to many different fields and with varied meanings. The classical idea of a keystone is attractive because it represents a top-down mechanism of ecosystem control that humans can potentially understand and manipulate (Busby et al., 2017; Trivedi et al., 2017; Hamonts et al., 2018). A few well-known macroecological examples in which keystones play important roles in stabilizing species diversity and ecosystem function are marine rocky intertidal zones dominated by the top predator starfish *Pisaster ochraceus* (Paine, 1966), riparian habitats dominated by extended *Populus* phenotypes (Whitham et al., 2003), kelp forests that disappear without otters (Estes and Palmisano, 1974), and the very landscape of Yellowstone National Park following the reintroduction of gray wolves (Ripple et al., 2001).

Interest in applying the keystone species concept to microbial communities and the plant microbiome has grown in recent years (Meyer and Leveau, 2012; Ze et al., 2012; Berry and Widder, 2014; Copeland et al., 2015). In the phytobiome, the concept of a keystone is attractive as a means of understanding how the plant controls its microbiome. Several authors have used computational network approaches and experimental methods to identify putative keystones. However, few studies have attempted to validate the role of putative keystones. Moreover, investigation of the mechanisms by which a plant recruits a keystone microbe for the purpose of manipulating its microbiome and larger ecosystem has only recently begun. Early studies show promising results and suggest that further research into keystone species recruitment would contribute significantly to the fields of sustainable agriculture, conservation, and ecological restoration.

### 2.1. Identification and Validation of Keystone Species

Identifying and validating keystone species is inherently complex. By definition, classical keystones increase alpha and beta diversity and stabilize ecosystem function via top-down mechanisms and are typically described as being rare or low in abundance, having few direct interactions with other community members. In the phytobiome, keystones are theorized to play vital roles in helping establish the core microbiome (Harrison et al., 2018). In contrast to members of the core microbiome, for which low-level taxonomic composition may vary but ensemble function is conserved (Lemanceau et al., 2017), keystones perform unique functions inherently tied to their identity. While core microbes also perform important functions for the host plant and its community, removal of a core member does not necessarily lead to dissolution of the community. The keystone's status as a community linchpin means its functional role and very presence are often difficult to separate from those of its community members—a “control” community without the keystone simply does not exist or function in any comparable fashion. Furthermore, identifying low abundance and rare microbes that exert top-down control or have an otherwise out-sized effect on community structure requires deep or single-cell sequencing of soil and plant samples (Oberholster et al., 2018). However, currently used statistical methods filter out low abundance taxa and those not present in the majority of samples to avoid biasing analyses (Berry and Widder, 2014; Agler et al., 2016; Duhamel et al., 2018; Oberholster et al., 2018), thus rare taxa recovered by deep sequencing are likely to be pruned a priori.

Some studies have taken the approach of focusing on organisms previously theorized to be keystones and then attempting to characterize the putative functional role they fulfill in the community (Agler et al., 2016; Duhamel et al., 2018), while others approach the problem from the opposite direction by seeking out rare organisms that could be candidates for carrying out well-studied functions of interest (Ze et al., 2012). These approaches essentially utilize the classical keystone concept to narrow the hunt for the needle in the haystack. While these studies clearly demonstrate that microbes do fulfill “classical” keystone roles, it is likely that a broader or more flexible definition will become necessary as research in this arena progresses; the “classical” keystone model was developed within the macroecological realm and therefore may not sufficiently encapsulate uniquely microbial keystone characteristics (Meyer and Leveau, 2012). This unexplored frontier means that attempting to find new keystone taxa—particularly in uncharacterized systems—is difficult because the characteristics and roles unique to microbial keystones are largely unknown at this stage. In contrast to description of keystones at the macroecological scale, recent studies have found evidence that microbial keystones may be ephemeral, performing a specific function in a particular environment or developmental stage, but playing a less prominent role in others (Duhamel et al., 2018; Oberholster et al., 2018). For example, many vertically transmitted microbes that are dispersed on seed surfaces (such as *Alternaria fulva* on *Astralagus lentiginosus* seeds) fulfill important roles in early holobiont development that affect the long-term composition of the phytobiome (Harrison et al., 2018).

Interest in using co-occurrence networks to identify putative microbial keystones has been rapidly growing (Berry and Widder, 2014; Copeland et al., 2015; Agler et al., 2016; Banerjee et al., 2018). Such networks are often created by calculating the correlation of pairwise OTU abundances. Several papers have used computational methods to identify network microbial “hubs” (OTUs that have a high number of connections within the community). However, hub taxa are not necessarily keystones (Berry and Widder, 2014; Rottjers and Faust, 2018). Using co-occurrence as a proxy for biologically meaningful interactions is problematic. Understanding microbiome interactions relies upon properly identifying the true sphere and scale in which interactions take place. The lack of directly observed interactions presents an immense challenge because the niches which partition true interaction from mere co-occurrence are an unknown prior and vary greatly, often in ways not understood by researchers. For example, the interior of a single leaf presents a multitude of ecologically distinct microhabitats. Among niches, microbes may have few or zero interactions but still “co-occur” because they are present in the same macrohabitat, i.e., the leaf (Berry and Widder, 2014; Hamonts et al., 2018). Nevertheless, in uncharacterized microbiomes, network methods can offer valuable insight regarding community dynamics and serve as a first step in the process of identifying the keystone taxa that serve as the linchpins in community structure.

### 2.2. Evidence for Plant-Driven Keystone Interactions

The majority of studies on microbial keystones have not investigated the mechanisms underpinning host interactions, perhaps because methods to explicitly identify and validate the effects of keystone species are still largely under development. However, there appears to be promising evidence that plants are actively recruiting keystone microbes. Using a combination of field experiments and network analyses to investigate the rhizosphere communities of sunflower and sorghum crops, Oberholster et al. (2018) identified 47 hub taxa and putative keystone microbes deemed to be necessary for maintaining network structure, including *Proteobacteria, Rhizoplanes, Flavisolibacter, Povalibacter, Nitrososphaera, Lysobacter*, and *Sphingomonas*. Although investigating plant-keystone-species interactions was outside the scope of the study, Oberholster et al. (2018) found that the abundance of taxa in the rhizosphere varied with soil chemistry and plant developmental stage. Furthermore, changes in soil chemistry were correlated with plant species and plant developmental stage. Phylogenetic diversity of the sorghum rhizosphere was significantly correlated with soil carbon and nitrogen concentrations, whereas sunflower rhizosphere diversity was correlated with potassium, calcium, magnesium, and phosphorus. Together they suggest that differences in plant root exudates likely contributed to the structure of rhizosphere communities, potentially through putative keystone taxa.

Despite the fact that formal investigation of keystone species is still in early days, mycorrhizal fungi have long been recognized as playing an essential role in structuring the microbiome of plant hosts. Both arbuscular mycorrhizal (AM) and ectomycorrhizal (ECM) fungi have been described as keystone species (Gamper et al., 2010; Soka and Ritchie, 2015; Oberholster et al., 2018), and much can be drawn from mycorrhizal fungal host interactions. Duhamel et al. (2018) found a strong association between plant species and rhizosphere communities in greenhouse and field experiments. In greenhouse experiments in which three plant species (*Pinus muricata, Baccharis pilularis*, and *Ceanothus thyrsiflorus*) were planted in media containing a tripartite mix of soils from each plant's native range (and thus each plant's native microbial community), the final composition of each plant's microbiome most strongly resembled that of each host plant's native range. The significant similarity in community structure between greenhouse and native soils supports the idea that plants can play an active role in selecting their microbiome structure. In agreement with Oberholster et al. (2018), Duhamel et al. (2018) found that community composition and keystone prominence changed over time. For example, members of the genera *Rhizopogon* and *Suillus* (both belonging to Suillineae) are keystone mutualists; these ECM basidiomycetes are abundant during development of *P. muricata* seedlings and under conditions similar to those that occur during range expansion, but are rare during other phases such as in well-established monodominant stands. Although the mechanism by which *P. muricata* recruits keystones such as *Suillus pungens* was not investigated by Duhamel et al. (2018), the obligate relationship of *Pinus* species with ECM has long been of interest. *Suillus* species in particular have been shown to facilitate *Pinus* invasions, as was demonstrated for *S. luteus* and *P. contorta* (Hayward et al., 2015). Kikuchi et al. (2007) found that *S. bovinus* germinated when co-cultured with *P. densiflora*, and could be induced to germinate in the absence of the host by treatment with flavonoids previously reported from root exudates, including hesperidin, morin, rutin, quercitrin, naringenin, genistein, and chrysin. Liao et al. (2016) found that relationships between members of *Pinus* and *Suillus* were species-specific, and that compatible *Pinus* and *Suillus* pairings elicited expression of unique gene sets including genes related to production of fungal small secreted proteins and host leucine-rich repeat-containing R proteins. Moreover, the JA and ET pathways were found to be upregulated during incompatible pairings (but interestingly, SA was not).

In contrast to beneficial keystones that increase microbial alpha and beta diversity (Herren and McMahon, 2018), pathogenic keystones tend to reduce diversity. Agler et al. (2016) found a significant correlation between the obligate biotrophic oomycete *Albugo laibachii* and host genotype in *Arabidopsis thaliana*. The authors found that phyllosphere alpha and beta diversity were dramatically reduced following infection. Similar to the effects of beneficial keystones, community structure was stabilized in infected hosts relative to pathogen-free hosts. Agler et al. (2016) did not investigate the mechanisms involved in susceptibility or resistance to *A. laibachii*; however, closely related *A. candida* has been shown to alter host metabolism in *A. thaliana* (Chou et al., 2000). Ruhe et al. (2016) showed that rather than killing the host or severely decreasing fitness, *A. laibachii* maintains a level of infection that is tolerable to the host. Additionally, there is evidence that *A. laibachii* is largely able to tolerate host defense mechanisms and suppresses only a small portion of host activity. Ruhe et al. (2016) reasoned that the immune tolerance is an adaptation over non-host evolved pathogens that would serve as competitors to *A. laibachii* or kill the host. Even though *A. laibachii* is classified as a pathogen due to predominantly negative effects on the host, leaving the host's defense response intact to compete against more virulent pathogens has a less negative impact on *A. thaliana*. Furthermore, the “keystone” role of *A. laibachii* is in stabilizing the post-infection community composition, which prevents other pathogens from invading while plant immunity is already compromised.

In consideration of the challenges associated with identifying and validating keystone species, it is not a surprise that there is a shortage of studies investigating plant-driven mechanisms for recruiting or maintaining relationships with keystones. However, some inferences can be made based on previous studies that investigated host mechanisms with regards to a single organism or a simple community (many of which will be described in subsequent sections and should influence the future direction of keystone research). Synthetic (constructed) communities offer a promising avenue for isolating host mechanisms (Bodenhausen et al., 2014). Niu et al. (2017) constructed a seven member synthetic community on maize roots and discovered through iterative removal of members that the community collapsed without *Enterobacter cloacae*. In addition, when the community was intact, it functioned to suppress *Fusarium verticillioides*. Niu et al. (2017) did not investigate the host mechanisms involved in these interactions, but we argue doing so is a natural next step that will yield valuable fundamental information. Although simplified communities are likely to miss some important host-microbe dynamics, they are a good place to start for gaining basic understanding. In addition, the use of microfluidics can facilitate the dissection of complex plant-microbe interactions by facilitating the fine-scale manipulation and imaging of real-time plant recruitment of and colonization by microbes (Massalha et al., 2017; Stanley and van der Heijden, 2017). Furthermore, microfluidic methods allow plant exudates, phytohormones, and internal signaling cascades to be characterized using proteomic, transcriptomic, and other techniques to gain new insight into host mechanisms that operate at specific spatial-scales. To date, these methods have largely been used to study culturable microbes. However, the use of ensemble culturing techniques such as those used by Agler et al. (2016) could facilitate study of unculturable and rare taxa, which would provide more nuanced and realistic insights into holobiont dynamics. Furthermore, the use of such techniques in combination with time-series experiments would inform how plant recruitment of keystones varies with developmental stage, which would in turn improve our understanding of the core microbiome changes over time.

## 3. Plant Defense Modulation of the Microbiome From a Host Gene and Phytohormone Perspective

Concepts such as evolutionary pressure and dynamical feedback have shaped our understanding of plant-microbe interactions. A recognized hypothesis of the plant immune system, referred to as the zigzag model, characterizes the defense response as a successive pattern-triggered immunity (PTI) and effector-triggered immunity (ETI), set of responses. PTI is viewed as the first line of defense and involves protein recognition receptors (PRRs) on the cell surface. PRRs bind often conserved microbial compounds referred to as microbe (pathogen) associated molecular patterns, or MAMPs (PAMPs). The binding of microbial compounds by PRRs then elicits a signal cascade of defense responses that inhibit microbial growth. ETI is the second line of defense, comprised of intracellular resistance (R) genes that contain nucleotide binding leucine rich repeat (NB-LRR) domains. Resistance genes code for proteins that bind microbial virulence effector proteins. Binding of the effector proteins then triggers a signal cascade that often results in cell death. PTI can be evaded by the microbe, eliciting successive ETI responses. ETI responses can also be evaded by the microbe, creating an evolution of responses by the plant and evasion by the microbe. However, the model views the PTI and ETI responses as distinct, and implicitly views the PTI responses as more conserved evolutionary than the ETI. The standard PTI-ETI model contradicts observations indicating that PRR may evolve similarly to R genes, and that certain R genes may play a similar role to PRR genes (Cook et al., 2015). The review by Cook et al. (2015) suggests that the plant immune system is an interacting set of co-evolving responses that occur both within and outside the cell, and is a response that involves multiple signal cascades. Phytohormones are a fundamental part of the resultant defense signal.

General defense related phytohormones form part of what is referred to as the plant's systematic acquired resistance (SAR) and induced systemic resistance (ISR) (Pieterse et al., 2012; Fu and Dong, 2013).

Of the various phytohormones, ethylene (ET), jasmonic acid (JA), and salicylic acid (SA) have been classically characterized in some plant defense role (Pieterse et al., 2012) and have been shown to preferentially impact certain bacterial phylla in a community (Carvalhais et al., 2014). There is an emerging interest in the potential of phytohormones to shape the plant's microbial community. Given the importance of the microbial community in plant defense, growth, and sustainability, it is therefore important to understand the hormone-microbial dynamic. We therefore describe recent evidence toward the role of ET, JA, and SA in shaping the microbiome community. Additionally, we look at research into the interplay of the respective hormone biosynthetic pathways and how they may assist in microbial colonization of the plant.

### 3.1. Ethylene

Originally shown in oats *Avena sativa* and broad bean, *Vicia faba*, the volatile hormone ET influences plant growth (Laan, 1934), with many further studies further characterizing the role of ET on plant growth and development (Burg and Burg, 1966; Smalle et al., 1997; Sukumar, 2010). The role of ET in plant defense was suspected due to a measured increased in ET biosynthesis during early PTI response in *Nicotiana tabacum* (Bailey et al., 1990; Sharon et al., 1993). It later became evident that ET signaling was required for the expression of the receptor kinase (FLS2) which binds bacterial flagelin (flg22) in *A. thaliana* and thereby triggers the defense response (Mersmann et al., 2010). ET has also been shown to be involved with stress tolerance (Thao et al., 2015). There has been emerging interest in characterizing the role that ET may play in not only defense from an individual microbe, but also in how ET influences the community (Nascimento et al., 2018). Mutant *A. thaliana* lines have provided an ideal framework to work toward characterization of ET.

A synthetic community approach was used in *A. thaliana* to determine host genetic factors that may influence phyllospheric bacterial community structure (Bodenhausen et al., 2014). Bodenhausen et al. found that ET-insensitive mutants, which possessed a mutation in the EIN2 gene, displayed a significant shift in the bacterial community structure at the genus level. They identified an increase in the relative abundance of *Variovorax*, a genus consisting of the metabolically diverse gram negative *Variovorax pardoxus* (Han et al., 2011). It is difficult to determine if increased abundance is directly associated with ET, or if it is mediated by pathway cross talk, especially given that Bodenhausen et al. observed a significant decrease in *Variovorax* abundance in the SA-insensitive mutant.

Another experiment involving *ein2* mutants also showed a shift in the rhizosphere bacterial community composition in non-autoclaved soil (Doornbos et al., 2011). However, the result was not observed in autoclaved recolonized soil. The recolonization of the autoclaved soil in particular consisted of either species that survived the autoclave process or those species in the surrounding environment, indicating that the initial microbial community composition may play a role in the capacity of ET to influence microbial structure. While the aforementioned hypothesis was not explored in Doornbos et al. (2011), they did observe supporting evidence in that bacterial community shifts were observed prior to disease symptoms, and no significant differences were observed in the absences of defense signaling. The latter indicates that a potential selective pressure triggering a defense response may be required to observe ET-mediated microbial community shifts. An early shift in community before disease symptoms may be justified in that ET is known as a potential early response signal (Mersmann et al., 2010). Therefore, an existing microbial community may provide the necessary pressure to elicit an ET response that can shape the community structure.

There is evidence to suggest that genotype effect on root microbiome is much weaker than the potential effect on the leaf microbiome (Wagner et al., 2016). While the full genotypic differences were not fully characterized in the Wagner et al. study, it was determined that leaves and roots differ in glucosinolate concentrations. Glucosinolate may be regulated by JA and ET signaling during rhizobacterial colonization (Pangesti et al., 2016). Therefore, glucosinolate secondary metabolites may provide a possible strategy for microbial community selection.

### 3.2. Jasmonic Acid

The role of JA in plant defense was first described as part of an infection-mediated wound response (Farmer and Ryan, 1992). Other associations to wound healing and herbivory-related defense have since been observed for components of the lipid-derived hormone's biosynthetic pathway (Li et al., 2005; Schilmiller et al., 2007; Koo et al., 2009; Christensen et al., 2013). JA has also been associated with plant necrotroph defense (Plett et al., 2014; Wei et al., 2016).

There is evidence to suggest that the phytohormone JA may have the capacity to shape the root microbial community by means of root exudates (Bertin et al., 2003; Sasse et al., 2018). In particular, evidence of root-associated allelopathic and chemotactic negative and positive selection for constituents of the microbiome has been discussed in Bais et al. (2004). *Arabidopsis thaliana* knock-out mutants *myc2* and *med25* were shown to have disrupted JA signaling pathways that result in attenuated wounding, herbivory, and defense responses as well as altered root exudate profiles (Carvalhais et al., 2015). Carvalhais et al. found correlations between specific exudate concentrations and the abundances of several bacterial microbes. While the roles of these exudate compounds in shaping the microbial community are not yet fully understood, compounds such as tryptophan and fructose are chemotactic to several bacteria (Ordal et al., 1979; Yang Y. et al., 2015). One way JA might facilitate host-driven selection of the plant microbiome is by fine tuning the concentrations of root exudates that attract various microbes.

Furthermore, a recent root exudate study in maize found benzoxazinoids, which is regulated by JA, exhibited the capacity to alter the composition of the microbial community (Oikawa et al., 2001; Hu et al., 2018). Here, Hu et al. also experimented with the effect of benzoxazinoid inoculation on soil, which identified improved herbivory defense, exhibited genotype dependent growth reduction, and increased levels of JA. It has previously been shown that benzoxazinoids are chemotactic for *Pseudomonas putida* KT2440, which elicit JA priming and thereby resistance to particular fungi (Neal et al., 2012; Neal and Ton, 2013). A differential secondary metabolite analysis of genotype root exudates in Monchgesang et al. identified differential concentration of glucosinolate, SA catabolites, and dihydrohydroxy JA, indicating JA associated genotypic influences on root exudation (Mönchgesang et al., 2016). JA's influence of root exudates may in turn influence the rhizosphere microbiota, given the strong correlation between genotype root exudation and the rhizosphere bacterial community structure (Micallef et al., 2009). However, direct experimentation is needed to test JA's influence and better understand the potential mechanisms involved.

### 3.3. Salicylic Acid

The role of SA in plant defense was first described in tobacco, against tobacco mosiac virus (White, 1979), where supplementation of diluted aspirin induced a defense response. SA has since been described as an important component in plant defense signaling (Shah, 2003). It is believed that SA forms part of the plant's defense strategy against biotrophes, as opposed to necrotrophes which are more associated with the JA and ET pathways (Glazebrook, 2005).

In its capacity to regulate the microbiome, SA has been shown to modulate the composition of the root microbiome at the family level in *A. thaliana* (Lebeis et al., 2015). *Arabidopsis* knockout mutant lines were used, where essential components of the SA, JA, and ET pathways were targeted. Expectedly, mutants with the three respective pathways knocked out showed a lower survival rate. Apart from the microbial compositional shift, it was shown that certain bacterial endophytic families may actually require SA-related processes to colonize. Exogenous supplementation of SA resulted in an observed altered microbial community profile indicating potential SA-mediated preferential selection for microbial families (Lebeis et al., 2015). Treating ginsing with phenolic acids over a six-year period resulted in rhizosphere fungal community shifts (Li et al., 2018). Li et al. observed dramatic relative abundance changes of taxa at both the genus and phylum levels, with SA-associated changes significantly different from control. However, a study in wheat observed no significant SA-induced root microbial diversity shifts in a 72 h time window (Liu et al., 2018). There is recent evidence examining the heterogeneity of the SA pathway, whereby in wheat the induction of SA may result in various different chemical and even physiological responses (Gondor et al., 2016). It is therefore unclear whether the underlining mechanism is temporal-, genotype-, or community-specific.

### 3.4. Cross-Talk and the Interplay Between Pathways

Given the potential capacity of ET, JA, and SA to modulate the microbiome, it is often unclear how much cross-talk there is between pathways; however, there is evidence of significant interplay between the respective defense pathways (Koornneef et al., 2008; Diezel et al., 2009; Song et al., 2014; Yang Y.X. et al., 2015). The interplay of phytohormones can be antagonistic, with microbes being able to exploit the antagonism to facilitate colonization and thus evade host defense responses (Jacobs et al., 2011; Plett et al., 2014; Jha et al., 2018). The microbes themselves may utilize effector molecules to actively manipulate the phytohormone pathways and elicit antagonism between the pathways (Kazan and Lyons, 2014). The classical interpretation of the interplay between JA, SA, and ET is reviewed in Yang Y.X. et al. (2015). Unfortunately, little has been done thus far in untangling the potential pathway interaction and their effect on the microbial community. Here we therefore discuss recent evidence that may suggest the capacity of pathway cross-talk to manipulate and shape the microbiome.

As indicated above in the study of Bodenhausen et al. (2014), a statistically significant difference was observed in *Variovorax* abundance in both the ET and SA associated *A. thaliana* knockout mutants. There is the potential that *Variovorax* abundance is actually managed by the interaction between these respective pathways, given that ET and SA generally have a positive interaction and that *Variovorax* has been shown to be positively correlated with SA (Badri et al., 2013; Yang Y.X. et al., 2015).

The phytohormone abscisic acid (ABA) is well-known in its role in drought stress, salinity stress and as a modulator of plant defense signaling. It has been shown to negatively impact the SA-associated defense pathway, both positively and negatively affect JA-associated defense, and has been shown to affect ET-associated pathogen defense (Pieterse et al., 2012; Takatsuji and Jiang, 2014). In potting soil, exogenous application of ABA has resulted in preferential selection for *Cellvibrio, Limnobacter*, and *Massilia* microbes at the genus level (Carvalhais et al., 2014). However, cross-talk between ABA and the other phytohormone defenses, in terms of the effect on whole microbial communities, is largely still unexplored. Additionally, the mechanism of ABA's affect on whole microbiomes is poorly understood. Certain microbial species have been shown to leverage ABA cross-interaction either by producing ABA or effecting ABA biosynthesis, thereby effecting plant-microbe dynamics (Jiang et al., 2010; Ho et al., 2013; Takatsuji and Jiang, 2014).

## 4. Abiotic Plant Stress and the Impact on Microbial Communities

Environmental stressors, including drought stress, temperature stress, and salinity stress, impact plant development, metabolic activity, and the ability for the plant to interact with its phytobiome. The altered phytohormonal signaling and community structure alters the plant's ability to resist stress, resist disease, and alters the capacity for nutrient acquisition (Hawkes and Connor, 2017). While many studies have been performed on the response of the microbiome to abiotic stress and the potential beneficial and deleterious effects on the host, it is less clear how the host influences its microbiome under abiotic stress conditions.

### 4.1. Drought Stress

Drought stress has a significant impact on plant growth, development, metabolism, and mortality (Allen et al., 2010). Changes in the host in response to drought, in addition to changes in environmental conditions, induce plant-specific (Naylor et al., 2017) and compartment-specific (Santos-Medellín et al., 2017) selection of microbial communities; however, many drought responses, including changes in the microbiome, are conserved across species (Naylor et al., 2017) and soil types (Santos-Medellín et al., 2017).

*Actinobacteria* is commonly enriched in drought across a wide range of different compartments and plant species (Naylor et al., 2017; Santos-Medellín et al., 2017; Garcia et al., 2018; Timm et al., 2018; Xu et al., 2018). In *Sorghum*, drought causes developmental delays in the root microbiome, selecting for monoderms (Xu et al., 2018). During drought, there was an association between increased carbohydrates in the roots and increased carbohydrate transporters in *Actinobacteria* (Xu et al., 2018), suggesting altered root metabolites may play a role in selecting certain species. Additionally, monoderms are less affected by the increase in ROS by the plant during drought stress, relative to diderms (Shade et al., 2012). Host ROS metabolism genes were shown to be associated with *Streptomyces* (a genus of *Actinobacteria*) in *Populus* leaves (Garcia et al., 2018), potentially showing a more universal drought association between the host and its phytobiome. ROS metabolism has been shown to be a general change across species, omics levels, and compartments in drought (Fang et al., 2015; Abraham et al., 2018; Garcia et al., 2018; Zandalinas et al., 2018) that has impacts beyond that of *Actinobacteria*.

ROS metabolism transcription and defense response transcription are correlated during drought with a variety of taxa including *Rhizophagus* and nematodes (Garcia et al., 2018). ROS have been shown to modulate the host microbiome, including mitigating nematode infection (Nath et al., 2017) in soybeans (Beneventi et al., 2013) and in tomatoes (Vos et al., 2013). ROS have also been show to be beneficial in regulating rhizobial symbiosis in *Medicago truncatula* (Andrio et al., 2013). In addition to ROS, other hormones with defense affecting properties, such as ABA, are able to alter the host microbiome. ABA is upregulated in drought in a variety of plants, including *Populus, Arabidopsis, Sorghum*, etc. (Daszkowska-Golec, 2016; Sah et al., 2016; Kalladan et al., 2017; Garcia et al., 2018). Upregulation of ABA is associated with increased disease susceptibility in a variety of plants (Xiong and Yang, 2003; Gao et al., 2016; Pye et al., 2018). However, the plant's ability to withstand disease under stress has been shown to be plant- and disease-specific (Sinha et al., 2016). Furthermore, combined drought and disease stress has been shown to have an increased ability to mitigate disease (Pandey et al., 2015).

Metabolite production and exudates of the plant, including carbohydrates, amino acids, and other nutrients, are altered in response to drought stress (Bouskill et al., 2016; Tripathi et al., 2016; Abraham et al., 2018; Timm et al., 2018). Under more mild drought conditions, rhizodeposition is increased, while under more severe drought conditions, rhizodeposition is decreased, causing the exudate profile to be related to the severity of the drought experienced (Preece and Peñuelas, 2016). The change in metabolite profile with the plant also correlates with changes in the bacterial community, with root community composition in *Arabidopsis* shown to be dependent on the exudate profiles of the host plant (Badri et al., 2013). Under drought, an increase in hydrolytic enzymes responsible for breaking down complex carbohydrates such as lignin, cellulose, and other plant metabolites within the microbial communities has been shown (Bouskill et al., 2016). Additionally, bacteria can alter ethylene production within the plant with ACC deaminase (Arshad et al., 2008), which in turn alters plant growth and metabolite profiles to the benefit of plants and microbes (Mayak et al., 2004; Zhang et al., 2018). Not only can the host plant alter its exudate profile to recruit organisms, the microbial community can influence what compounds are being exuded, potentially creating a reciprocal relationship between the community and exudate profile. It is currently unknown how much of the exudate profiles are a plant-driven process and how much the microbial community can influence that process.

### 4.2. Temperature and Salinity Stress

Temperature stress can often accompany drought stress and has an impact on the fluidity of plant membranes (Sangwan et al., 2002), plant metabolism (Koscielny et al., 2018), ROS activation (Kotak et al., 2007), and protein misfolding (Scharf et al., 2012). Under colder temperatures, root nodulation is decreased in beans, lentils, and peas (Junior et al., 2005), and many organisms that live in the nodules have lower survivability (Singh et al., 2012). Under higher temperatures, a plant is less able to combat pathogens (Mendes et al., 2011), allowing for colonization of disease-causing organisms. However, some of the disease suppression lost during high heat can be associated with the loss of microorganisms that naturally inhibit plant diseases (van der Voort et al., 2016). Conversely, in a wheat high temperature seedling plant, high temperatures induce a WRKY transcription factor that promotes resistance to *Puccinia striiformis* infection (Wang et al., 2017).

In addition to drought and temperature stress, salinity stress also limits the types of plants that can grow in a given area. High salinity can cause ionic and osmotic stress that limits plant growth and damages plant cells (Zhu, 2002). Also, under salinity stress, plants have increased ROS generation, ABA synthesis, and accumulation of carbohydrates within the plant (Gupta and Huang, 2014), resulting in a similar response to water stress. Plants also respond similarly to drought in that ACC deaminase also can confer salinity resistance to the host plant (Qin et al., 2014). Many studies have been performed that have identified potential plant growth promoters under high salinity stress (Yuan et al., 2016; Hussain et al., 2018; Fouda et al., 2019). For example, 14 halotolerant microbes were shown to improve canola root growth under salt stress by decreasing ethelyne production (Siddikee et al., 2010). Additionally, *Piriformospora indica* was correlated with an increase in barley antioxidants under salt stress (Baltruschat et al., 2008). Salt tolerance can also be promoted by fungi, such as *Montagnulaceae* potential improving nitrogen availability in *Suaeda salsa* under salt stress (Yuan et al., 2016). Despite many studies identifying potential plant growth promoters under saline stress, there is limited knowledge of host plants influencing selection of community structure under salinity stress.

## 5. Remaining Challenges

It is clear that there are recent efforts toward identifying plant specific modes of action to control its microbiome. However, in a number of cases it is challenging to determine whether any mode of action was plant-mediated, microbial-mediated, or environment-mediated. Furthermore, the complex and potentially reciprocal nature of interaction adds to the challenge of identifying plant control mechanisms, which is evident when identifying and understanding phytohormone-based control mechanisms. Phytohormone pathways are interconnected, and they mediate abiotic stress responses in complex and sometimes antagonistic manners. The multi-layer interplay must then be delineated to understand the plant-microbe dynamic. Apart from the possible plant-associated complexity, there is the dynamic among the microbial community that must also be understood. Individual taxa in a microbiome may have a large effect on the whole microbiome structure, and the plant-based mechanisms may effect influential members.

### 5.1. Toward Understanding Combinatorial Plant Mechanisms

Diverse plant accession libraries provide a good framework to begin to compartmentalize and understand the dynamic of combinatorial interactions. For example, the *A. thaliana* mutant lines have provided a wealth of information on plant mechanisms that may influence microbial populations; however, they do not address the potential for confounding interactions between pathways. Additionally, the mutant lines on their own do not address the question of whether or not a respective pathway is necessary for the observed microbial shift. Approaches that use the supplementation of plant associated compounds identifying interplay between pathways and microbes need to be further addressed. Mutant lines with a combination of potential pathways effected, or general quantitative trait loci (QTL) studies, may be able to address the interaction confounding factors. Unfortunately, using model organisms such as *Arabidopsis* may not provide generalized results to all species given the variety in hormone pathways and physiological-based mechanisms that plants have for interacting with their microbial communities. There can be significant difference in the phytohormonal pathways between plant species (De Vleesschauwer et al., 2014), with the differences being associated with the biosynthetic pathway itself or the associated function of the pathway (Gondor et al., 2016). Additionally, certain plant species may be more amenable to hormonal amendment than others (De Vleesschauwer et al., 2014). It would therefore be prudent to investigate other, non-model, organisms in a variety of environments with the above mentioned techniques. Furthermore, multiple aspects of a study would need to be manipulated, such as hormone abundance, abiotic stressors, biotic stressors, and presence or absence of a microbial community members (including putative keystone species), to elucidate potential combinatorial effects between genotype, hormones, stress, and microbes.

### 5.2. Approaches to Unravel Microbial Community Dynamics

Synthetic or constructed communities provide an efficient approach to model microbial diversity. In contrast to axenic controls that allow for the ability to hypothesize about the colonization capacity or pathogenicity of an individual microbe, synthetic communities allow for the discovery of higher level interactions between plants and the microbial community. To therefore begin to understand the complex ecosystem may require an ecosystem point of view, including those based upon constructed communities in controlled environments (http://eco-fab.org/). However, synthetic communities may not be able to capture all of the complex site to site variation observed in natural environments, given that site variation can be a dominant factor in microbial diversity for certain plants (Whitaker et al., 2018). Nevertheless, constructed communities provide a model from which we can begin to reason, hypothesize, and understand the plant's role in dynamic microbial community interactions.

Using computational biology approaches in combination with experimental field and lab methods, including tools such as microfluidics and constructed communities will help advance understanding regarding plant recruitment of keystone microbes. Further understanding of host-mediated recruitment of its microbiome will in turn improve our ability to effectively and efficiently construct or manipulate plant-microbe systems for improved agricultural and ecological restoration efforts.

### 5.3. The Holobiont

A significant body of literature focuses on the soil or rhizosphere; however, we know that other compartments, including the root microbiota, can also influence above-ground phenotypes (Pangesti et al., 2017). Root exudates have a reciprocal impact on the microbial community and are influenced by the abiotic stress, biotic stress, and phytohormones. Some drought stresses cause irreversible changes to root exudates (Gargallo-Garriga et al., 2018), which can be important when trying to engineer a community to promote plant growth under a variety of environmental conditions. Therefore, it is important to study plant-mediated effects on other compartments, but determining if effects are compartmental cross-talk, abiotic stress, or direct plant associated is still an open problem.

## Author Contributions

PJ, AF, and BG wrote the manuscript. DJ and GT edited the manuscript and provided guidance on content.

### Conflict of Interest Statement

The authors declare that the research was conducted in the absence of any commercial or financial relationships that could be construed as a potential conflict of interest.
